# Detection of ALK Gene Rearrangements in Non-Small Cell Lung Cancer by Immunocytochemistry and Fluorescence in Situ Hybridization on Cytologic Samples

**DOI:** 10.5146/tjpath.2021.01542

**Published:** 2022-01-21

**Authors:** Suneel Rachagiri, Parikshaa Gupta, Nalini Gupta, Manish Rohilla, Navneet Singh, Arvind Rajwanshi

**Affiliations:** Department of Pathology, Post Graduate Institute of Medical Education and Research, Chandigarh, India; Department of Cytology and Gynaecologic Pathology, Post Graduate Institute of Medical Education and Research, Chandigarh, India; Department of Pulmonary Medicine, Post Graduate Institute of Medical Education and Research, Chandigarh, India

**Keywords:** Lung adenocarcinoma, Non-small cell lung carcinoma, Anaplastic lymphoma kinase, ALK rearrangement, Fluorescence in situ hybridization, Immunocytochemistry, D5F3

## Abstract

*
Objective:* Determination of the molecular status is mandatory for personalized treatment of patients with non-small cell lung carcinoma. The present study was performed to detect anaplastic lymphoma kinase (*ALK*) rearrangements in pulmonary adenocarcinoma on cytology samples, using immunocytochemistry (ICC) and fluorescence in situ hybridization (FISH) on cell-blocks to assess the diagnostic reliability of these two techniques.

*
Material and Method:* A total of 50 confirmed lung adenocarcinoma cases were included. In all the 50 cases, ICC was performed for ALK protein expression by using the D5F3 clone on Ventana platform. On the basis of ALK protein expression on ICC, the cases were categorized as ALK positive (2+ or 3+ strong cytoplasmic granular positivity) or negative (negative or 1+ cytoplasmic granular positivity). FISH for detection of *ALK* gene rearrangement was performed in 7 ALK ICC positive cases and 7 ALK ICC negative cases using the Vysis *ALK* break apart FISH probe kit.

*
Results:* Based on ICC, 7(14%) cases were ALK positive and 43(86%) were ALK negative. ALK gene rearrangements in lung adenocarcinoma were more commonly seen in non-smokers (31.25%) as compared to smokers (6.25%). Among the ALK-ICC positive cases, FISH demonstrated break apart signal in 5 cases (ALK- ICC positive); however, no break-apart signals were seen in 2 ALK-ICC positive and all the seven ALK-ICC negative cases.

*
Conclusion:* Immunocytochemistry on cell- blocks using DF53 clone is a highly sensitive and specific method for the detection of ALK gene rearrangements in lung adenocarcinoma with a greater number of ALK positive cases being detected on ICC as compared to the ALK-FISH.

## INTRODUCTION

The treatment for non-small cell lung carcinoma (NSCLC) has become personalized with the advancements in molecular pathology and identification of specific therapeutic target molecules (1). A variety of molecular abnormalities have been recognized in lung cancer including mutations in Kirsten rat sarcoma viral oncogene homolog (KRAS), epidermal growth factor receptor (EGFR), BRAF, MEK and HER2 and phosphatidylinositol 3-kinase (PI3K) pathway oncogenes. ALK, ROS1 and RET showed structural rearrangements which come up with novel therapeutic targets. MET and fibroblast growth factor receptor 1 (FGFR1) amplification is noted in adenocarcinoma and SCC respectively (2).** **EGFR mutations are seen in around 32.3% of lung adenocarcinoma cases (3). *EGFR* mutations, like, point mutations in exons, and 21 and exon 19 deletions, are associated with a dramatic therapeutic response to EGFR tyrosine kinase inhibitors (TKI) (4). The molecular methods used for detection of EGFR mutations include Sanger sequencing (SS), Next generation sequencing (NGS) and polymerase chain reaction-based methods (5).

Anaplastic lymphoma kinase (ALK), is a tyrosine kinase receptor, encoded by the *ALK* gene. *ALK *gene rearrangements are seen in 1.9-6.8% cases of NSCLCs (6). The most common genetic rearrangement involves the echinoderm microtubule associated protein-like 4 (*EML4*) and *ALK* leading to formation of the *EML4-ALK* fusion gene that encodes for a chimeric protein with intrinsic tyrosine kinase activity. However, fusion genes involving other partners have also been detected. Identification of the *ALK *gene rearrangement is a mandatory diagnostic test for NSCLC patients (7,8). This is mainly owing to the availability of effective ALK-inhibitors like crizotinib, alectinib, and ceritinib, which lead to good therapeutic response and better five-year survival rates as compared to the standard chemotherapy regimens (9). Currently, three methods are available for detecting *ALK* gene rearrangements: fluorescence in situ hybridization (FISH), immunohistochemistry (IHC) and real-time PCR (RT-PCR). FISH has been considered the gold standard method for detecting *ALK* gene rearranged NSCLC. However, the recent guidelines recommend that IHC, using FDA approved antibodies, is an equivalent alternative for ALK testing (8). Although ALK testing is frequently performed on histopathological tissues, testing using cytologic samples is sparsely documented. The present study was undertaken to detect *ALK* gene rearrangements by using immunocytochemistry (ICC) and the FISH technique on cell-blocks, in cases diagnosed as lung adenocarcinoma on cytology samples.

## MATERIALS and METHODS

This was a one-year prospective study performed on a total of 50 lung adenocarcinoma (ADC) cases diagnosed on fine needle aspiration cytology (FNAC) or pleural fluid cytology. The study was approved by the Institute Ethics Committee (NK/4423/MD). The objectives were to detect *ALK* gene rearrangements by immunocytochemical (ICC) staining using the D5F3 clone and FISH technique on cell-blocks and to compare the clinicopathologic characteristics amongst the ALK positive and ALK negative cases.

Direct and/or sediment smears were prepared from cytologic samples (both air-dried and 95% ethanol-fixed) and rest of the cytologic material was rinsed into a glass tube containing 1 ml of 1% ammonium oxalate for cell-block preparation. The air-dried smears were stained with May Grunwald Giemsa (MGG) and the wet-fixed smears with haematoxylin and eosin (H&E) and/or Papanicolaou stain. The cell-blocks were prepared using an already standardized plasma clot method. ICC was performed on the cell-blocks, wherever needed, to subtype the tumors using TTF1, p40, CK7 and Napsin A.

### ALK IHC Using D5F3 Clone

The Ventana ALK (D5F3) CDx assay was used for the detection of ALK protein expression as a surrogate marker for ALK gene rearrangements. The strength of cytoplasmic granular positivity was graded as 3+ (strong positivity in >90% tumor cells); 2+ (moderate cytoplasmic granular positivity in 20-90% tumor cells); 1+ faint cytoplasmic positivity in <20% cells; and 0 (negative for cytoplasmic positivity).

### ALK Gene Rearrangement by FISH

FISH was performed for detection of *ALK* gene rearrangements in ICC- positive and equal number of randomly selected ICC- negative cases. FISH was performed using the Vysis *ALK* break apart FISH probe kit (Abbott Molecular). Fluorescence signals (ALK 5’ probe (Spectrum Green) and the ALK 3’ probe (Spectrum Orange)) were recorded after viewing under fluorescence microscope (Olympus WX63 Epi-illumination fluorescence microscope). *** *** At least 50 tumor cell nuclei were evaluated for each case and the positivity was taken as nuclei showing split signals or deleted signals (presence of single orange signal) (Table 1). A tumor was interpreted as ***negative*** if less than 5 out of 50 tumor cells (<5/50 or <10%) were positive. A tumor was interpreted as ***positive*** if 25 cells out of 50 tumor cells (>25/50 or >50%) were positive. A tumor was interpreted as ***equivocal*** if 5-25 cells (10 to 50%) were positive. For ***equivocal*** cases, a second unbiased evaluation of slide by another cytopathologist was performed, following which both the first and second cell counts/readings were added together and a final percentage was calculated for 100 cells. If the average percentage of the positive cells was <15% (<15 positive nuclei/100 evaluated tumor nuclei), the sample was interpreted as negative. However, if the average percentage of the positive tumor cells was >15% (>15 positive nuclei/100 tumor cell nuclei evaluated), the sample was interpreted as positive.

**Table 1:** Various signal patterns observed on fluorescence in situ hybridization and their interpretations.

**Signal arrangements in the tumor cells**	**Interpretation**
Two signals of the same color and size, separated by a distance less than two signal diameters were recorded as one signal	**Negative for** ***ALK*** **rearrangement**
Fused orange and green signals. The signals are either overlapping, adjacent, or less than two signal diameters apart	
A single green signal without a coexisting orange signal in addition to fused and/or broken apart signals indicates a deletion of the orange region of the ALK probe and is taken as negative. The target area (tyrosine kinase domain) of the drug is located within the area targeted by the orange probe	
**Signal arrangements in the tumor cells**	**Interpretation**
These nuclei contain rearranged or “broken apart” signals, two or more signal diameters apart	**Positive for** ***ALK*** **rearrangement**
More than one set of broken apart signals can be seen in one nucleus	
Fused signal(s) and broken apart signal(s) can be seen in one nucleus	
A single orange signal (deleted green signal) in addition to fused and/or broken apart signals can be seen in one nucleus	
Fused signals, broken apart signals and deletions can be seen in the same nucleus	

### Statistical Analysis

For data analysis, SPSS (version 22.0) software was used. The Shapiro-Wilk test was applied to check the normality of continuous data like age. For normally distributed data, mean and SD were reported. Categorical variables like gender, smoking status, pathologic diagnoses, stage, etc. were reported as frequency and percentage. The independent t-test was used to compare the mean of normally distributed quantitative variables between two groups (ALK IHC positive and negative). The chi square/Fisher’s Exact test was applied to find out any association between categorical variables and the study groups. A *p value* of < 0.05 was taken as significant.

## RESULTS

**Figure 1:**
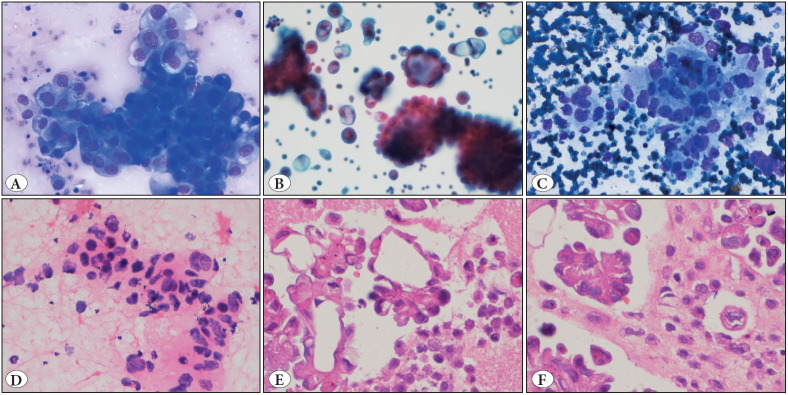
**A,B)** Pleural fluid smears showing papillary, three dimensional clusters of tumor cells with moderate nuclear pleomorphism, vesicular chromatin, prominent nucleoli and moderate to abundant amount of vacuolated cytoplasm (a: MGG, 40x; b: Papanicolaou, 20x); **C,D)** Fine needle aspiration smears showing loose clusters of tumor cells with moderate nuclear pleomorphism, vesicular chromatin, prominent nucleoli and moderate cytoplasm (c: MGG, 40x; b: H&E, 40x); **E,F)** Sections from the cell-blocks prepared from malignant pleural effusions showing acinar as well as papillary arrangement of the tumor cells (H&E; 40x)

A total of 50 primary lung adenocarcinoma diagnosed on the basis of cytomorphology and appropriate panel of immunocytochemical markers (TTF1, p40, Napsin A and CK7), were included in the study. Of these, 17 cases were reported as adenocarcinoma and 10 as NSCLC, favouring adenocarcinoma on FNAC. Another 23 cases were reported as metastatic lung adenocarcinoma in pleural effusion samples (Figure 1A-D). The age of the patients ranged from 28-82 years with the mean age being 57.5 years (standard deviation=11.1). The male:female ratio was 1.6:1 with 31 males and 19 females. The lesions were more common in the right lung (n=36) than the left lung (n=14) and the upper lobe was more commonly involved as compared to the lower lobe of the lung. The majority of the cases (n=23; 48%) in the present study were in TNM stage IV with 14 cases having evidence of extra-thoracic metastatic disease, mostly to the central nervous system and bone.

### Detection of ALK Gene Rearrangements by ICC

**Figure 2:**
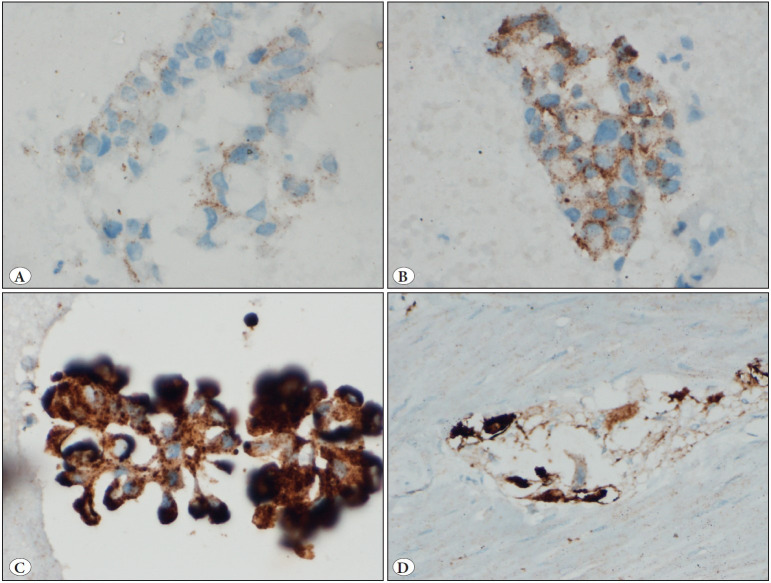
Panel of photomicrographs showing variable intensities of ALK immunocytochemistry (D5F3 clone) on cell-block sections: **A)** Weak and fine granular cytoplasmic staining (1+) in the tumor cells; **B)** Faint, coarse to fine granular cytoplasmic staining (2+) in the tumor cells; **C)** Strong, coarse granular cytoplasmic staining (3+) in the tumor cells; **D)** Section from the appendix (control tissue) showing coarse granular cytoplasmic staining (3+) in the ganglion cells (20x).

Seven *** *** (14%) cases showed cytoplasmic granular positivity for ALK antibody (D5F3 clone). Based on the staining intensity, 5 (71.4%) cases were categorized as 3+ ALK positive and two (28.6%) cases were categorized as 2+ (Figure 2A-D).

Clinicopathologic parameters were compared amongst ALK positive and ALK negative groups on ICC. ALK gene rearrangements were more frequently seen in females (4/7 (57.1%) cases being ALK positive) as compared to males (3/7 (42.9%) cases being ALK positive); however, this difference was not found to be statistically significant (p=0.08). The mean age of the patients in the ALK positive group (56 years) as compared to that of ALK negative group (59 years) was also not statistically significant (p=0.6).

Out of 48 cases with known smoking status, 32 (66.6%) were smokers and 2/32 (6.25%) cases were ALK positive. Among smokers, 26 (81.2%) were males and 6 (18.7%) were females. Among 16 (33.3%) non-smokers, 5/16 (31.25%) were ALK positive. It was seen that non-smokers were significantly associated with *ALK* gene rearrangements (p=0.03). Furthermore, it was more commonly detected in females with non-smoking status; however, this was not statistically significant (p=0.08).

Pleural effusion was noted in 20 (41.7%) cases; however, the presence of pleural effusion was not found to be significantly associated with *ALK* gene rearrangements (p=0.10). ALK positivity was seen more commonly in cases reported as adenocarcinoma as compared to cases reported as NSCLC, favour adenocarcinoma; however, this was not statistically significant (p=0.36) (Table 2).** **Furthermore, *ALK* gene rearrangements were seen in cases having focal solid and acinar (n=5; 71.4%), and papillary (n=2; 28.5%) architecture (Figure 1E,F).

**Table 2:** Distribution of various clinicopathological parameters among the ALK immunocytochemistry (ICC) and fluorescence in situ hybridization (FISH) positive and negative cases in the present study.

**Clinicopathological parameters**	**ALK ICC** ** ** **Positive, n=7 (%)**	**ALK ICC** ** ** **Negative, n=43 (%)**	***P*** **Value**	***ALK*** **FISH Positive, n=5 (%)**	***ALK*** **FISH Negative, n=9 (%)**
**Males**	3 (42.9)	28 (65.1)	0.40	2 (40)	3 (33.3)
**Females**	4 (57.1)	15 (34.9)		3 (60)	6 (66.7)
**Smokers**	2 (28.6)	30 (69.8)	0.03	1 (20)	7 (77.8)
**Non-smokers**	5 (71.4)	11 (25.6)		4 (80)	2 (22.2)
**Pleural effusion present**	5 (71.4)	15 (34.9)	0.10	5 (100)	0 (0)
**Pleural effusion absent**	2 (28.6)	28 (65.1)		0 (0)	9 (100)
**Adenocarcinoma**	5 (71.4)	12 (27.9)	0.36	2 (40)	1 (11.1)
**NSCLC, Adenocarcinoma**	1 (14.3)	9 (20.9)		1 (20)	0 (0)
**Metastatic Adenocarcinoma**	1 (14.3)	22 (51.2)		2 (40)	8 (88.9)

In addition, EGFR gene mutational analysis was performed by real time polymerase chain reaction in 46 (92%) cases and no case in the ALK positive group showed known mutations in the exons 18, 19, 20 and 21 of EGFR gene, reiterating the mutually exclusive existence of these genetic alterations.

Two out of seven ALK positive cases received Crizotinib or Ceritinib. One patient had progression of the disease and the other one showed a partial response.

### Detection of ALK Gene Rearrangements by FISH

**Figure 3:**
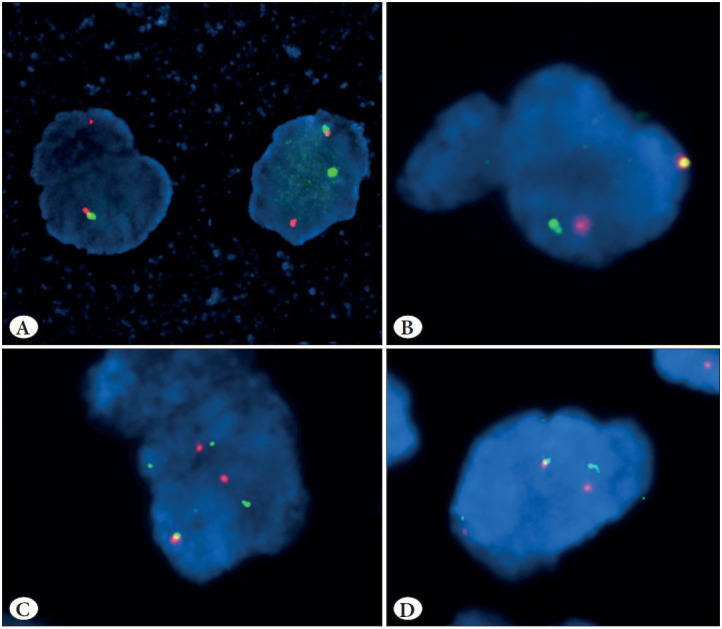
Panel of photomicrographs showing tumor cells with ALK rearrangement on FISH: **A)** Two tumor nuclei have a single orange signal (deleted green signal) in addition to fused signal; **B-D)** The tumor nuclei contain rearranged or “broken-apart” signals and fused signals.

**Figure 4:**
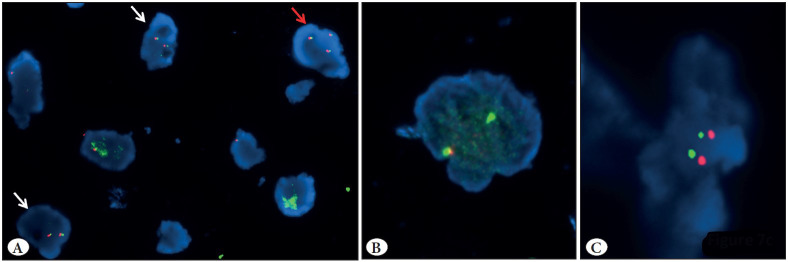
Panel of photomicrographs showing tumor cells negative for ALK rearrangement on FISH: **A)** Three tumor cells with non-rearranged signals, in the form of fused orange and green signals. Two cells show two fused signals (white arrows) and one cell shows three fused signals (red arrows), indicating a triploid tumour cell; **B)** A cell with a single green signal without a corresponding orange signal, in addition to a fused signal indicates a deletion of the orange portion of the ALK probe (considered negative); **C)** Non-rearranged cell with orange and green signals, which are less than two signal diameters apart.

FISH testing for *ALK* gene rearrangements using the Vysis *ALK* break-apart FISH probe kit (Abbott Molecular) was performed in a total of 14 randomly selected cases (7 ALK ICC positive cases and 7 ALK ICC negative cases). *ALK* rearrangements by the FISH technique could be detected in 5/7 (71.4%) cases, which were positive on ALK ICC. Among the *ALK*-FISH positive cases, the mean percentage of *ALK*-FISH positive rearranged nuclei was 79.25% (range 67-91%). FISH positive cases showed presence of split signal pattern (n=3) and combined 3’ deletion and split signal pattern (n=2)** **(Figure 3A-D). In addition, all the 7 ALK ICC negative cases were negative on *ALK* testing by FISH, indicating a good concordance between ICC and FISH (Figure 4A-C).

## DISCUSSION

*ALK *gene rearrangements are seen in 1.9-6.8% cases of NSCLCs (6).** **The most common *ALK *gene rearrangement in NSCLC is paracentric inversion on the short arm of chromosome 2, juxtaposing the 5’ end of EML4 (echinoderm microtubule associated protein-like 4) gene to the 3’ end of the *ALK *gene (10). This leads to the formation of *EML4-ALK *fusion gene, encoding for a chimeric protein with intrinsic tyrosine kinase activity. In addition to this, other break-apart and fusion partners may also be involved in *ALK* rearrangements.

There are 4 methods for detecting these genetic rearrangements including immunohistochemical staining (IHC), fluorescence in situ hybridization (FISH), reverse transcriptase‐PCR (RT‐PCR) and next generation sequencing (NGS). All of these methods have their own advantages and disadvantages. FISH is considered as the gold standard for detecting ALK rearrangements, however, well-validated IHC has been accepted as an equivalent alternative (7,8). NGS can detect all kinds of fusions, whereas, FISH and IHC provide no fusion specification and RT-PCR provides information only regarding *EML4-ALK* fusion (11).

In the present study, detection of *ALK* gene rearrangements in lung adenocarcinoma cases was carried out using ICC on cell-blocks. The mean age of the patients in our study is similar to that observed in the previous studies (12–15).

Lung adenocarcinoma is more common in smokers than in non-smokers. Similarly, in our study 66.6% patients were smokers and 33.3% were non-smokers. However, ALK gene rearrangements were seen more frequently in non-smokers (31.25%), which correlates well with some previous studies (10–15). The tumors were more common in the right lung (n=35) in the present study, which is similar to a previous study (16,17). Presence of pleural effusion was found to be higher in patients with *ALK* gene rearrangement as seen in the previous studies; however, this was not statistically significant (18,19). There was no statistically significant difference between lung cancer stage and *ALK* gene rearrangements which is in agreement with the previous studies (20).** **A thorough comparison of the present study with previously published studies for detection of *ALK* rearrangements is shown in the Table 3 (21–25). The prevalence of ALK rearrangements using FISH and IHC observed in the present study is in concordance with other studies wherein the prevalence ranged from 3 to 14.9% and 4 to 15.4%, for FISH and IHC, respectively.** **The concordance rates of ALK IHC and *ALK*-FISH in the published literature are variable and range from 75-100% (Table 3).** **The concordance rate between ICC and FISH in the present study was 66.7%.

**Table 3:** Comparison of the present study with previously published studies for detection of ALK rearrangements.

**Authors**	**Sample type**	**Preparation used**	**Number of samples**	**Methods**	**ALK + cases %**	**Concordance rate between IHC & FISH**
**Zhou et al. 2015 **(21)	MPE	CB	52	FISH ICC (D5F3) RT-PCR	9.6 15.4 13.5	100%
**Liu et al. 2015 **(22)	MPE	CB	66	FISH ICC (D5F3) RT-PCR	3 4.5 4.5	75%
**Wang et al. 2015 **(23)	MPE	CB	63	FISH ICC (D5F3)	10.3 12.7	100%
**Rosenblum et al. 2014 **(14)	FNA, MPE, BB	CB, DS, TP	71	FISH ICC (D5F3)	4.2 4	100%
**Minca et al. 2014 **(24)	FNA, MPE, BB, PE	TP CB	230 154	FISH ICC (D5F3)	7.8 6.5	98.7%
**Zhang et al. 2015 **(25)	FNA	DS, CTT	47	FISH ICC (D5F3)	14.9 10.6	93.6%
**Rogers et al, 2015 **(15)	TMA	Histopathology tissue	324	FISH IHC (D5F3)	1.2 1.5	80%
**Mok et al, 2021 **(13)	Histopathologic and cytologic samples (type not specified)		242	IHC/ICC FISH	242 203	83.9%
**Present study**	FNA, MPE	CB	50	FISH ICC, (D5F3)	35.7 14	66.7%

**FNA:** Fine needle aspiration, **MPE:** Malignant pleural effusion, **PE:** Pleural effusion, **BB:** Bronchial brushing, **TMA: **Tissue microarrays, **CB:** Cell-block, **FISH:** Fluorescence in situ hybridization, **RT-PCR:** Real-time polymerase chain reaction, **DS:** Direct smears, **CTT:** Cell transfer technique, **TP:** ThinPrep, **ICC:** Immunocytochemistry, **IHC:** Immunohistochemistry.

Higher ALK positivity rates with immunochemistry can be explained by the fact that ALK IHC detects the ALK protein expression but not the genetic changes. Similarly, lower positivity rates of ALK-FISH on cell-blocks, can be due to the presence of yet unknown type of *ALK* rearrangement or genetic abnormalities other than *ALK* rearrangements, which may be missed on FISH. As FISH is considered the gold standard test, the results may indicate that ALK-IHC had false-positive results. Similarly, a few authors have found that FISH can miss a good number of patients with *ALK-EML4* rearrangements who might benefit from targeted ALK therapy, so they strongly recommended ALK-IHC (19,26). When analysed alone with FISH, their cohort had 4 (7.8%) positive cases whereas the true incidence was 7 (13.7%) cases (19). This can be because of extremely minimal splitting of red and green signals giving false negative results. However, rare *ALK* translocations that do not cause over expression of ALK protein may lead to negative IHC and positive FISH results.

## CONCLUSIONS

*ALK* gene rearrangements in lung adenocarcinoma are more commonly seen in females, non-smokers and in patients having pleural effusions. Among the architectural patterns, *ALK* gene rearrangements were common in cases having focal solid, acinar and papillary architecture. Immunocytochemistry on cell-blocks using the DF53 clone is a highly sensitive and specific method for detection of ALK gene rearrangements in lung adenocarcinoma with greater number of ALK positive cases being detected on ICC as compared to *ALK*-FISH.

## Conflict of Interest

The authors declare no conflict of interest.

## Funding

None
